# Ultrasound-guided continuous erector spinae plane block vs continuous thoracic epidural analgesia for the management of acute and chronic postthoracotomy pain: a randomized, controlled,double-blind trial

**DOI:** 10.1097/PR9.0000000000001106

**Published:** 2023-11-07

**Authors:** Ehab Hanafy Shaker, Mamdouh Mahmoud Elshal, Reham Mohamed Gamal, Norma Osama Abdallah Zayed, Samuel Fayez Samy, Raafat M. Reyad, Mohammed H. Shaaban, Abd Alrahman M. Abd Alrahman, Ahmed Salah Abdelgalil

**Affiliations:** a Department of Anesthesia, Intensive Care, and Pain Management, National Cancer Institute, Cairo University, Giza, Egypt; b Department of Diagnostic & Interventional Radiology, Faculty of Medicine, Cairo University, Giza, Egypt; c Department of Surgical Oncology, National Cancer Institute, Cairo University, Giza, Egypt

**Keywords:** Analgesia, Erector spinae, Epidural, Thoracic

## Abstract

**Introduction:**

Postthoracotomy pain (PTP) is a severe pain complicating thoracic surgeries and its good management decreases the risk of PTP syndrome (PTPS).

**Objectives:**

This randomized controlled study evaluated the efficacy of ultrasound-guided continuous erector spinae plane block (ESPB) with or without dexmedetomidine compared with thoracic epidural analgesia (TEA) in managing acute postoperative pain and the possible emergence of PTPS.

**Methods:**

Ninety patients with chest malignancies planned for thoracotomy were randomly allocated into 3 equal groups. Group 1: TEA (20 mL of levobupivacaine 0.25% bolus, then 0.1 mL/kg/h of levobupivacaine 0.1%), group 2: ESPB (20 mL of levobupivacaine only 0.1% bolus every 6 hours), and group 3: ESPB (20 mL of levobupivacaine 0.25% and 0.5 μg/kg of dexmedetomidine Hcl bolus every 6 hours).

**Results:**

Resting and dynamic visual analog scales were higher in group 2 compared with groups 1 and 3 at 6, 24, and 36 hours and at 8 and 12 weeks. Postthoracotomy pain syndrome incidence was higher in group 2 compared with groups 1 and 3 at 8 and 12 weeks, whereas it was indifferent between groups 1 and 3. The grading system for neuropathic pain score was higher in group 2 compared with groups 1 and 3 at 8 and 12 weeks, whereas it was indifferent between groups 1 and 3. Itching, pruritis, and urine retention were higher in group 1 than in ESPB groups.

**Conclusion:**

Ultrasound-guided ESPB with dexmedetomidine is as potent as TEA in relieving acute PTP and reducing the possible emergence of chronic PTPS. However, the 2 techniques were superior to ESPB without dexmedetomidine. Erector spinae plane block has fewer side effects compared with TEA.

## Abstract 2

This study evaluated the effect of continuous erector spinae plane block on both acute and chronic postthoracotomy pain, and it was safe and effective.

## 1. Introduction

Lung cancer represents approximately 13% of cancer patients and accounts for a high percentage of cancer-related mortality.^9^ Lung resection is still the main therapeutic rationale. Thence, the count of chest procedures and postthoracotomy pain syndrome (PTPS) continuously rises. Postthoracotomy pain syndrome prevalence could reach 30% to 50%.^21^ The pain of PTPS is described as moderate to severe in one-fourth of cases and follows both open and video-assisted thoracotomies. It could impair sleep, the patient's psyche, activities of daily living, and overall quality of life (QOL).^30^ Postthoracotomy pain (PTP) may arise from the rib–periosteal–intercostal nerve complex, yet injury of the intercostal nerve is the main concern in emerging neuropathic PTPS.^24,47^ Postthoracotomy pain syndromes has several components, the neuropathic one being the cornerstone with myofascial and other pain generators.^31,32^

The most critical risk factor for developing PTPS is severe unrelieved postoperative pain (ie, pain evokes pain). Therefore, several locoregional and neuraxial techniques have been tried and implemented in multimodal analgesia for that purpose, including thoracic epidural analgesia (TEA), paravertebral block (PVB) besides serratus anterior plane block (SAPB), and recently erector spinae plane block (ESPB).^32,46^

Postthoracotomy pain syndrome is pain that recurs or persists at the thoracotomy scar at least 2 months after the surgery without infection or cancer recurrence.^38^Several interventional modalities have been described for treating this type of refractory pain syndrome, such as intrapleural analgesia, intercostal blocks, TEA using steroids, PVB, thoracic sympathectomies, radio frequency therapy of dorsal ganglia, intrathecal drugs delivery, and spinal cord stimulation.^1,16^ Recently, locoregional analgesic techniques have been successfully used to manage PTPS, including ESPB.^11,13^

Thoracic epidural analgesia and thoracic paravertebral block (TPVB) are the most used techniques for analgesia after thoracic surgery.^34^ However, TEA has several adverse effects, such as hypotension, motor blockade, hematoma, and abscess. Thoracic paravertebral block has a chance of epidural spread and pneumothorax, and multiple injections are needed if more than 4 dermatomal analgesics are required.^48^

Recently, ESPB was reported as a treatment for thoracic neuropathic pain.^26^ Erector spinae plane block is a relatively simple technique with easily identified sonographic landmarks, and a catheter is easily inserted into the plane after distention induced by the injection. In addition, the ESPB has the potential to provide both somatic and visceral sensory blockade.^26^

The selectivity of dexmedetomidine to the α_2_-receptors is 8 times of its prototype, clonidine. Accordingly, dexmedetomidine has more powerful sedative and analgesic effects than clonidine, with fewer hemodynamic derangements because of α_1_-receptor activation.^6^ Dexmedetomidine has been primarily used for sedation in intensive care settings. The unique analgesic properties of dexmedetomidine have encouraged anesthesiologists to use it perineurally. Previous studies have declared that dexmedetomidine potentiates local anesthetic effects when administered by the neuraxial route.^14^ This study aims to evaluate the efficacy of ultrasound-guided continuous ESPB with or without dexmedetomidine compared with TEA in managing acute postoperative pain and possible emergence of PTPS.

## 2. Methods

This prospective, randomized, controlled, double-blinded study was conducted in Egypt from Mach 2021 to June 2022. The study was approved by the local institutional review board (IRB Number: 201920015.2P) and registered at clinicaltrials.gov under the registry (NCT04531553). The study was conducted following the Helsinki Declaration. After explaining the procedure and obtaining written formal consent, we included 90 patients of both sex scheduled for thoracotomy for thoracic cancer surgeries under general anesthesia (lobectomy, pneumonectomy, and decortication) with the American Society of Anesthesiologists physical status classification I and II, age ≥18 and ≤65 years, and body mass index (BMI) between 20 and 40 kg/m^2^. The exclusion criteria were patient refusal, known sensitivity or contraindication to local anesthetics or dexmedetomidine, history of psychological disorders, localized infection at the site of block, and uncorrectable coagulopathies (platelet count below 50,000 or an INR > 1.5).

All included patients were randomly divided into 3 equal comparable groups using computer-generated random numbers; each included 30 patients.

Group 1 (n = 30): patients preoperatively received a TEA at the level T5 and T6 with a bolus of 20 mL of levobupivacaine (Chirocaine 0.5%, Abbott, Norway) 0.25%, then levobupivacaine 0.1% infused at a rate of 0.1 mL/kg/h until chest tube removal (usually done at 5–6 days post operation).

Group 2 (n = 30): patients preoperatively received US-guided ESPB on the same side, that is, scheduled operative side, the puncture point of the skin was infiltrated with 2% lidocaine, and once the structures were identified with ultrasound at the level of T5 transverse process, a bolus of 20 mL of levobupivacaine 0.25% was injected on the deep aspect of erector spinae muscle with catheter insertion. A 20 mL of bolus of levobupivacaine 0.1% was injected every 6 hours until chest tube removal.

Group 3 (n = 30): with the same procedure as group 2, the patients received a bolus of 20 mL of levobupivacaine 0.25% and 0.5 μg/kg of dexmedetomidine Hcl (Precedex, Hospira, Inc, Rocky Mount, NC) on the deep aspect of erector spinae muscle, then catheter insertion; 20 mL bolus of levobupivacaine 0.1% with dexmedetomidine 0.5 μg/kg was injected every 6 hours until chest tube removal.

All patients were assessed the day before surgery in a preoperative visit to evaluate their medical status and laboratory investigations and to fulfil all the inclusion and exclusion criteria mentioned above. The patients were instructed on how to report pain using the visual analog scale (VAS) score rating, in which 0 = “no pain” and 10 = “worst possible pain.”

### 2.1. The technique of thoracic epidural

With aseptic conditions under LA, the epidural space was identified(T5-T6 in midline approach) in sitting position using 18-G Tuohy needle by the loss of resistance to air technique. Then, an 18-G Portex epidural catheter was inserted 5 cm beyond the needle tip and the needle was removed. After checking the patency of the catheter, the catheter was fixed with a fixator, and 2 mL of 0.25% levobupivacaine was given as a test dose. Then, the patient placed in the supine position then another 18 mL of 0.25% bupivacaine was given.

### 2.2. The ultrasound-guided erector spinae plane block

**Figure 1.**
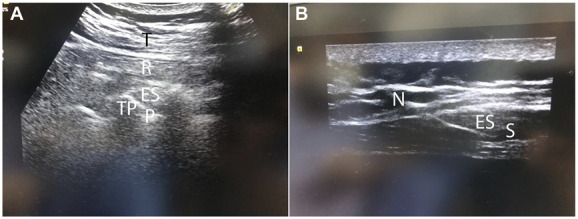
(A) Ultrasonography of pleura (P), transverse process (TP), trapezius, erector spinae (ES), and rhomboid major muscles (R). (B) Ultrasonography showing R-X Coudé needle (N) in contact with the transverse process underneath ES muscle after injection of saline (S).

**Figure 2.**
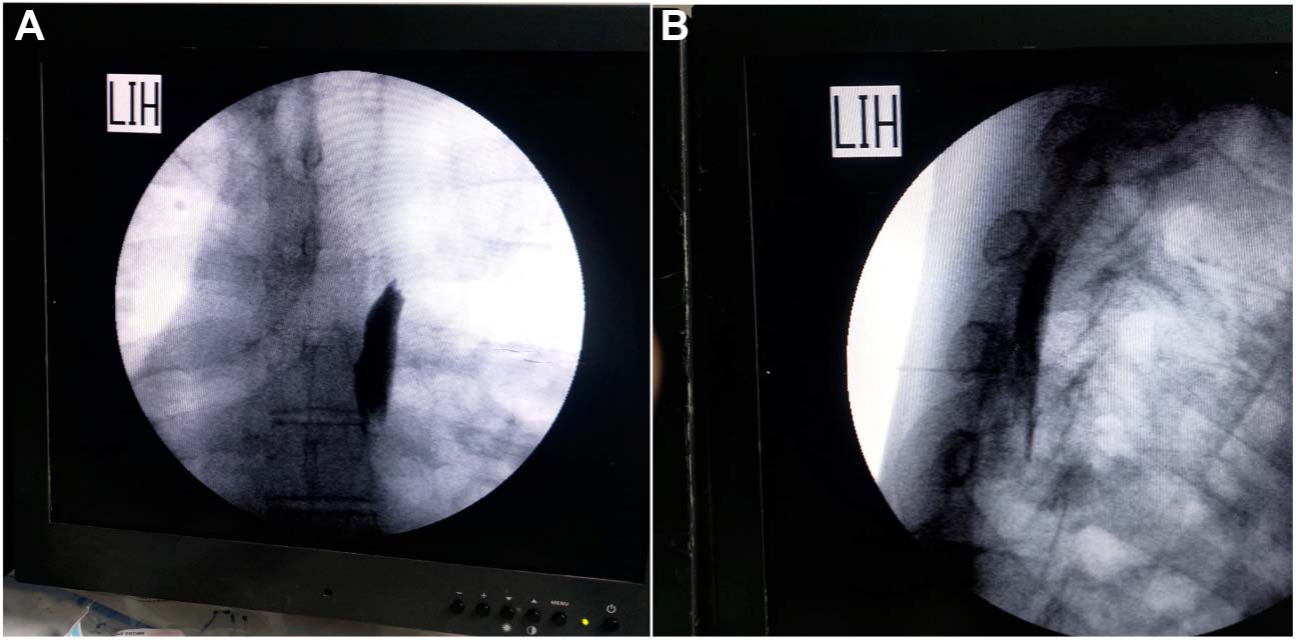
(A) P/A fluoroscopic view showing R-X Coudé needle with the RACZ catheter threaded after contrast injection; (B) Lateral fluoroscopic view showing R-X Coudé needle with the RACZ catheter threaded after the cranial and caudal spread of contrast.

Ultrasound measurements were performed using a Sonosite Edge (SonoSite, Inc., Bothell, WA) machine and an HFL38X linear transducer (Sonosite Inc) 13 to 16 MHZ.^11^ The patient was placed in a sitting position under complete aseptic condition, and a cover sheath was used for the ultrasound probe with an appropriate amount of lubricating gel applied on the probe, a high-frequency, linear, ultrasound transducer placed in a longitudinal orientation 3 cm lateral to the T5 spinous process. This should reveal 3 superficial muscles to the hyperechoic transverse process shadow: trapezius, rhomboid major, and erector spinae. The skin was anesthetized using 3 mL of 2% lidocaine. A 17‐G, 89‐mm R‐X Coudé epidural needle (Epimed, Johnstown, NY) was inserted in plane in a cephaled-to-cauded direction to place the tip into the fascial plane on the deep (anterior) aspect of erector spinae muscles, and 20 mL of levobupivacaine 0.25% or a mixture of 20 mL of levobupivacaine 0.25% with 0.5 μg/kg of dexmedetomidine was injected. The injectate included 5 mL of contrast medium iohexol 180 mg I/mL (Omnipaque, Nycomed, Oslo, Norway) (Fig. 1A, B). The location of the needle tip was confirmed by visible fluid spread, lifting the erector spinae muscle off the bony shadow of the transverse process. A reinforced radiopaque epidural catheter (19 G, 14 inches) (BREVI‐KATH Epimed) (RACZ catheter) was threaded and verified by a brief P-A fluoroscopy (Fig. 2A, B).

The success of the block was confirmed by the loss of pinprick sensation on the dermatomal site of the block after 30 minutes of injection, and patients with a failed block were excluded.

### 2.3. Anaesthesia management

Premedication with 0.02 mg/kg of midazolam was administered intravenously 30 minutes preoperatively. All included patients were monitored continuously using electrocardiography, noninvasive blood pressure, peripheral oxygen saturation, and end-tidal carbon dioxide—using the Datex-Ohmeda S5 anesthesia monitor, model no: USE1913A—throughout the surgical procedure. Induction of anesthesia was performed for all groups using a regimen of 2 μg/kg of IV fentanyl, 2 mg/kg of propofol, and 0.5 mg/kg of rocuronium. Anesthesia was maintained with inhaled sevoflurane with MAC 2% to 2.5% in oxygen-enriched air (FiO_2_ = 50%), and top-up doses of IV rocuronium (0.1 mg/kg) were administered as required. All patients received 1 gm of IV paracetamol. Additional bolus doses of 0.5 µg/kg of fentanyl were given if the mean arterial blood pressure or heart rate rose above 20% of baseline levels and calculated. Patients were mechanically ventilated at appropriate settings that keep end-tidal CO_2_ at 30 to 35 mm Hg. One reading of mean arterial pressure and heart rate was taken before induction of general anesthesia to be defined as a baseline reading and then regularly recorded immediately before surgical incision and at 30-minute intervals intraoperatively. By the end of the surgery, the residual neuromuscular blockade was reversed using Sugammadex (2 mg/kg), and extubation was performed after complete recovery of the airway reflexes.

All patients were transferred to the postanesthesia care unit where pain scores (VAS), MAP, and heart rate were recorded immediately on arrival, then at 2, 4, 6, 12, and 24 hours postoperatively. The patients were observed in the postoperative care unit for 2 hours, and rescue analgesia was provided as IV morphine 0.1 mg/kg per dose (max 0.3 mg/kg/d) boluses if the pain score was >3. The total amount of morphine given in 24 hours was recorded in the 3 groups. After that, the patients were shifted to the ward, and IV acetaminophen 1 g every 8 hours was prescribed.

Any adverse effects such as nausea, vomiting, hypotension, bradycardia, oversedation, or hematoma were recorded. Postoperative nausea and vomiting (PONV) were rated on a four-point verbal scale^3^ (none = no nausea, mild = nausea but no vomiting, moderate = vomiting one attack, severe = vomiting > one attack); 0.1 mg/kg of IV ondansetron was given to patients with moderate or severe postoperative nausea and vomiting.

#### 2.3.1. Measurement tools

All measured data were collected by an on‐duty ICU resident unaware of the study.

(1) Duration of the procedure, ICU stay, total hospital stay duration, pathology, lung resection procedure (metastasectomy, wedge resection, lobectomy, pneumonectomy, decortication, pleuropneumonectomy), baseline quality of life scale (QOLS), and pre/postoperative chemo/radiotherapy.
(2) VAS score was the primary outcome; it is a horizontal 10‐cm line with zero on the left end indicating no pain and 10 cm on the right end indicating the worst imaginable pain. The average VAS was assessed every 6 hours in the first 24 hours, and then, the average VAS was assessed daily in the first week (both at rest VAS [VAS‐R] and the VAS during coughing or movement, that is, the dynamic VAS [VAS‐D]). The average VAS values were also assessed at 2, 3, 4, 8, and 12 postoperative weeks.
(3) Assessment of the possible emergence of PTPS (PTPS incidence) at weeks 8 and 12. The neuropathic PTPS cases were screened using the grading system for neuropathic pain 0 means NO, 1 means possible, 2 probable, and 3 definite positive. Patients with a neuropathic component grade 2 (probable) or 3 (definite) are considered having PTPS.^10,39^

All patients requiring pain therapy were followed up at the pain clinic. Patients with neuropathic thoracic pain were treated according to the local institutional protocol. From the second postoperative week, pain therapy was offered in the form of adjuvant therapy (pregabalin 75–300 mg/d and/or amitriptyline 10–25 mg/d) plus analgesics “paracetamol or NSAIDs (VAS < 40 mm), tramadol HCl 100 to 600 mg/d (VAS: 40–70 mm), and oxycodone 20 to 80 mg/d (Targin tablets, Mundipharma, 20 mg oxycodone/10 mg naloxone) (VAS ≥ 70 mm).”

(4) Quality of life was assessed using the Flanagan QOLS, a 16‐item (domain) questionnaire with each item scored from 1 to 7 points. The scale was explained to the patients by the pain physician, and the total score was calculated and recorded at the preoperative assessment (baseline) and postoperative weeks 2, 3, 4, 8, and 12.^19^
(5) The activity level was assessed using the Barthel activities of daily living scale (ADL) at postoperative weeks 2, 3, 4, 8, and 12. This scale comprises 10 basic daily activities (bowel, bladder, feeding, toilet, bathing, dressing, grooming, walking, stairs, and transfer), with each item scored as 0 = need complete help, 1 = need some help, or 2 = need no help.^18^
(6) The incidence of adverse events was recorded in all groups.
(7) Intraoperative and postoperative opioid consumption was recorded in all groups.

### 2.4. Sample size calculation

The sample size calculation was done by G*Power 3.1.9.2 (Universitat, Kiel, Germany). We performed a pilot study (5 cases in each group), and we found that the mean (±SD) of resting VAS at 12 hours was 2.4 ± 1.52 in group 1, was 3.4 ± 0.55 in group 2, and was 2.6 ± 1.34 in group 3. The sample size was based on the following considerations: 0.378 effect size, 95% confidence limit, 80% power of the study, 1:1 group ratio, and 18 cases were added to each group to overcome dropout. Therefore, we recruited 90 patients in each group.

### 2.5. Statistical analysis

Statistical analysis was performed by SPSS version 25 (IBM Inc, Chicago, IL). Shapiro–Wilks normality test and histograms were used to test the distribution of quantitative variables to select accordingly the type of statistical testing: parametric or nonparametric. Parametric variables were expressed as mean and standard deviation (SD) and were compared using the analysis of variance among the 3 groups with post hoc (Tukey) test to compare every 2 groups. Nonparametric variables (eg, VAS) were expressed as the median and interquartile range (IQR) and were analyzed using the Kruskal–Wallis test; Mann–Whitney (*U*) test was performed to compare every 2 groups. Categorial variables were expressed as frequency and percentage and were statistically analyzed by χ^2^ test. A 2-tailed *P* value of ≤0.05 was considered statistically significant.

## 3. Results

**Figure 3.**
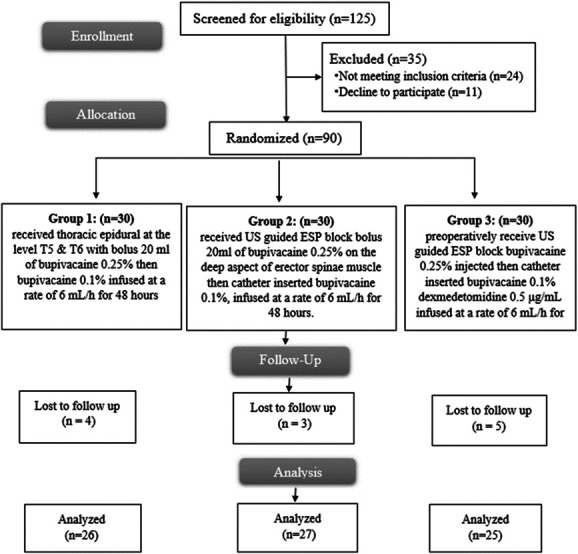
Consort flow diagram of the participants through each stage of the trial. ESP, erector spinae plane.

In this trial, 125 cases were assessed for eligibility. Ninety patients were allocated into 3 equal groups. Of these, 4 patients in group 1, 3 in group 2, and 5 in group 3 dropped out during follow-up (Fig. 3).

Baseline characteristics and therapy showed no significant difference between the groups (Table 1).

**Table 1:** Baseline characteristics and therapy of the studied patients.

	Group 1 (n = 26)	Group 2 (n = 27)	Group 3 (n = 25)	*P*
Sex				0.877
Male	18 (60%)	17 (56.67%)	16 (53.33%)	
Female	8 (26.67%)	10 (33.33%)	9 (30%)	
ASA				0.099
ASA II	16 (53.33%)	14 (46.67%)	8 (26.67%)	
ASA III	10 (33.33%)	13 (43.33%)	17 (56.67%)	
Age (y)	47.27 ± 9.96	47.41 ± 11.78	50.14 ± 9.78	0.773
Weight (kg)	63.35 ± 5.11	62.19 ± 4.25	62.33 ± 4.43	0.604
Height (m)	1.63 ± 0.07	1.63 ± 0.07	1.61 ± 0.08	0.415
BMI (kg/m^2^)	23.72 ± 1188.26	23.3 ± 846.31	23.98 ± 624.16	0.738
Therapy				
Chemotherapy	9 (30%)	11 (36.67%)	6 (20%)	0.556
Radiotherapy	7 (23.33%)	5 (16.67%)	6 (20%)	
Lung resection procedures				0.8
Mastectomy	2 (6.67%)	1 (3.33%)	5 (16.67%)	
Wedge resection	1 (3.33%)	1 (3.33%)	1 (3.33%)	
Pleuropneumonectomy	3 (10%)	3 (10%)	4 (13.33%)	
Pneumonectomy	4 (13.33%)	6 (20%)	3 (10%)	
Decortication	142.48 ± 11.32	138.9 ± 13.43	143.67 ± 13.03	
Lobectomy	2.52 ± 0.51	2.62 ± 0.5	2.57 ± 0.51	
Duration of procedure	142.48 ± 11.32	138.9 ± 13.43	143.67 ± 13.03	0.15
ICU stay (d)	2.52 ± 0.51	2.62 ± 0.5	2.57 ± 0.51	0.558
Hospital stays (d)	6.1 ± 0.89	5.86 ± 0.91	6.05 ± 0.86	0.497

Data are presented as mean ± SD or frequency (%).

ASA, American Society of Anesthesiologists; BMI, body mass index; ICU, intensive care unit.

Visual analog scale at rest was significantly higher in group 2 compared with groups 1 and 3 at 6, 24, 36 hours and at 8 and 12 weeks and showed no significant difference between groups 1 and 3 at these measurements. Visual analog scale showed no significant difference between the groups at preoperative, 12, 18, 30, 42, 48 hours, third day, fourth day, fifth day, seventh day, and at 2, 3, and 4 weeks (Table 2).

**Table 2:** Visual analog scale score at rest in the studied groups.

	Group 1 (n = 26)	Group 2 (n = 27)	Group 3 (n = 25)	*P*	
Preoperative	2.5 (2–3.75)	3 (1.5–4)	3 (2–4)	0.556	
6 h	2 (1–3)	3 (2.5–4)	3 (1–3)	0.001	*P*1= <0.001 *P*2 = 0.358 *P*3 = 0.012
12 h	3 (2–3)	3 (2–4)	3 (1–4)	0.687	
18 h	2 (1.25–3)	3 (1–4)	2 (2–3)	0.826	
24 h	3 (2–4)	5 (4–5)	2 (1–3)	<0.001	*P*1 = 0.03 *P*2 = 0.051 *P*3= <0.001
30 h	2 (2–3)	2 (2–4)	2 (1–3)	0.531	
36 h	2.5 (2–3.75)	5 (3.5–5)	2 (1–3)	<0.001	*P*1= <0.001 *P*2 = 0.568 *P*3= <0.001
42 h	2.5 (2–3)	3 (1–3)	3 (2–4)	0.613	
48 h	3 (1.25–4)	2 (1–3)	3 (2–4)	0.188	
3 d	1 (0–1.75)	1 (0–1)	1(0–2)	0.604	
4 d	1 (0–2)	1 (0–2)	1 (1–2)	0.899	
5 d	1 (0–2)	1 (0–1)	1 (0–1)	0.559	
6 d	1.5 (1–2)	1(0–2)	1 (0–2)	0.675	
7 d	1 (0.25–1.75)	1 (0.5–2)	1(0–1)	0.147	
2 wk	1 (0–2)	2 (0.5–2)	1 (0–2)	0.369	
3 wk	1 (0–1.75)	1 (0–2)	1 (0–2)	0.408	
4 wk	1 (0.25–2)	1 (0–1)	1 (1–2)	0.261	
8 wk	0.5 (0–1)	4 (1–6)	0 (0–1)	0.001	*P*1 = 0.001 *P*2 = 0.974 *P*3 = 0.001
12 wk	1 (0.25–1)	2 (1–6)	0 (0–1)	<0.001	*P*1 = 0.007 *P*2 = 0.262 *P*3= <0.001

Data are presented as median (IQR), *P*1: between groups 1 and 2, *P*2: between groups 1 and 3, *P*3: between groups 2 and 3.

Visual analog scale at movement was significantly higher in group 2 compared with groups 1 and 3 at 6, 24, 36 hours and 8, and 12 weeks but showed no significant difference between groups 1 and 3 at these measurements. Visual analog scale showed no significant difference between the groups at preoperative, 12, 18, 30, 42, 48 hours, third day, fourth day, fifth day, seventh day, and 2, 3, and 4 weeks (Table 3).

**Table 3:** Visual analog scale score at movement in the studied groups.

	Group 1 (n = 26)	Group 2 (n = 27)	Group 3 (n = 25)	*P*		
6 h	2 (2–3.75)	4 (3.5–5)	3 (2–4)	<0.001		*P*1 = <0.001 *P*2 = 0.475 *P*3 = 0.001
12 h	3 (2–4)	3 (2.5–4)	3 (2–4)	0.605		
18 h	3 (2–4)	3 (2–4)	3 (2–4)	0.674		
24 h	3.5 (2.25–4)	5 (4–5)	3 (2–3)	<0.001		*P*1 = 0.002 *P*2 = 0.088 *P*3 = <0.001
30 h	3 (3–4)	3 (2–4)	3 (2–4)	0.334		
36 h	3 (2–4)	6 (5–7)	3 (2–4)	<0.001		*P*1 = <0.001 *P*2 = 0.588 *P*3 = <0.001
42 h	3 (2–3.75)	3 (2–4)	3 (2–4)	0.542		
48 h	3(2–4)	2 (1–3.5)	3 (2–4)	0.108		
3 d	1 (0–1.75)	1 (0.5–2)	1 (0–2)	0.531		
4 d	1 (1–2)	1 (0–1.5)	1 (0–2)	0.481		
5 d	1 (0–2)	1 (0–2)	1 (1–2)	0.652		
6 d	1 (0–1)	1 (0–1)	1 (0–2)	0.511		
7 d	1 (0–2)	1 (0–2)	1 (1–2)	0.767		
2 wk	1 (0–2)	1 (0–1)	1 (0–2)	0.982		
3 wk	1 (1–2)	1 (0–2)	1 (0–1)	0.222		
4 wk	1 (0–2)	1 (0–2)	1 (1–2)	0.594		
8 wk	1 (1–2)	2 (1–5)	1 (0–2)	0.033		*P*1 = 0.062 *P*2 = 0.516 *P*3 = 0.012
12 wk	1 (1–2)	2 (1–5)	1 (1–1)	0.025		*P*1 = 0.255 *P*2 = 0.118 *P*3 = 0.007

Data are presented as median (IQR). *P*1: between groups 1 and 2, *P*2: between groups 1 and 3, *P*3: between groups 2 and 3.

Postthoracotomy pain syndrome incidence was significantly higher in group 2 compared with both group 1 and group 3 at 8 and 12 weeks and showed no significant difference between groups 1 and 3.

The grading system for neuropathic pain score was significantly higher in group 2 compared with both group 1 and group 3 at 8 and 12 weeks, whereas it showed no significant difference between groups 1 and 3 (Table 4).

**Table 4:** Postthoracotomy pain syndrome and grading system for neuropathic pain in the studied patients.

	Group 1 (n = 26)	Group 2 (n = 27)	Group 3 (n = 25)	*P*		RR (95% CI)
PTPS						
8 wk	4 (15.38%)	14 (51.85%)	5 (20%)	0.007	*P*1 = 0.008 *P*2 = 0.727 *P*3 = 0.036	0.296 (0.112–0.784) 0.769 (0.232–2.540) 2.592 (1.092–6.152)
12 wk	3 (11.54%)	13 (48.15%)	4 (16%)	0.004	*P*1 = 0.006 *P*2 = 0.703 *P*3 = 0.019	0.239(0.077–0.744) 0.721(0.179–2.903) 3.009 (1.129–8.016)
Grading system for neuropathic pain						
8 wk						
No Probable Definite	21 (80.8%) 5 (19.2%) 0 (0%)	14 (51.9%) 7 (25.9%) 3 (12%)	21 (84%) 3 (12%) 1 (4%)	0.018	*P*1 = 0.021 *P*2 = 0 0.476 *P*3 = 0.038	
12 wk						
No Probable Definite	22 (84.6%) 3 (11.5%) 1 (3.8%)	15 (55.6%) 5 (18.5%) 7 (25.9%)	22 (88%) 0 (0%) 3 (12%)	0.027	*P*1 = 0.043 *P*2 = 0.137 *P*3 = 0.019	

Data is presented as median (IQR) and frequencies; PTPS, postthoracotomy pain syndrome; P1: between groups 1 and 2, P2: between groups 1 and 3, P3: between groups 2 and 3.

Intraoperative fentanyl consumption was significantly higher in group 2 compared with group 3 (*P* value = 0.019) and showed no significant difference between group 1 and groups 2 and 3. Postoperative morphine consumption was significantly higher in group 2 compared with group 1 and group 3 (*P* value =<0.001, <0.001 respectively) and showed no significant difference between groups 1 and 3 (Table 5).

**Table 5:** Intraoperative and postoperative opioids consumption in the studied groups.

	Group 1 (n = 26)	Group 2 (n = 27)	Group 3 (n = 25)	*P*	
Intraoperative fentanyl (µg/kg)					
Mean ± SD Median (IQR)	7.69 ± 15.64 0 (0–0)	16.67 ± 13.89 30 (0–30)	6 ± 13.09 0 (0–0)	0.014	*P*1 = 0.059 *P*2 = 0.777 *P*3 = 0.019
Postoperative morphine (mg/kg/d)					
Mean ± SD Median (IQR)	12.19 ± 9.41 0 (0–0)	25.15 ± 7.53 30 (0–30)	11.68 ± 7.59 0 (0–0)	<0.001	*P*1= <0.001 *P*2 = 0.921 *P*3= <0.001

Data are presented as mean ± SD, *P*1: *P* value between groups 1 and 2, *P*2: *P* value between groups 1 and 3, *P*3: *P* value between groups 2 and 3.

Quality of life showed no significant difference between the groups at baseline and at 2, 3, and 4 weeks. Quality of life at 8 and 12 weeks was significantly higher in both group 1 and group 3 compared with group 2 and showed no significant difference between groups 1 and 3 (Table 6).

**Table 6:** Quality of life scale baseline at postoperative weeks 2 to 12 weeks

	Group 1 (n = 26)	Group 2 (n = 27)	Group 3 (n = 25)	*P*	
Baseline	51.31 ± 18.68	40.41 ± 6.78	57.44 ± 22.19	0.316	
2 wk	62.65 ± 24.95	60.89 ± 23.65	70.96 ± 25.4	0.383	
3 wk	68.54 ± 25.45	56.07 ± 23.02	71.04 ± 24.31	0.081	
4 wk	61.65 ± 25.15	58.11 ± 22.13	69.52 ± 25.53	0.335	
8 wk	84.46 ± 18.08	63.56 ± 27.56	84.76 ± 19.9	0.004	*P*1 = 0.049 *P*2 = 0.321 *P*3 = 0.003
12 wk	84.54 ± 16.73	64.44 ± 24.07	85.44 ± 16.95	<0.001	*P*1 = 0.001 *P*2 = 0.805 *P*3= <0.001

Data are presented as mean ± SD, *P*1: *P* value between groups 1 and 2, *P*2: *P* value between groups 1 and 3, *P*3: *P* value between groups 2 and 3.

Activity level showed no significant difference between the groups at 2, 3, and 4 weeks. The activity level at 8 and 12 weeks was significantly higher in group 1 and group 3 compared with group 2 and showed no significant difference between groups 1 and 3 (Table 7).

**Table 7:** Activity level in the studied patients.

	Group 1 (n = 26)	Group 2 (n = 27)	Group 3 (n = 25)	*P*	
2 wk	8.68 ± 7.79	10.17 ± 5.61	12.58 ± 5.55	0.317	
3 wk	9.33 ± 7.44	8.02 ± 6.42	12 ± 5.99	0.181	
4 wk	7.24 ± 6.88	9.37 ± 5.62	9.96 ± 6.33	0.242	
8 wk	15.85 ± 2.8	11.52 ± 6.27	15.96 ± 2.49	0.012	*P*1 = 0.011 *P*2 = 0.946 *P*3 = 0.011
12 wk	16.69 ± 2.66	11.04 ± 6.51	15.17 ± 2.97	0.002	*P*1 = 0.001 *P*2 = 0.171 *P*3 = 0.038

Data are presented as mean ± SD, *P*1: *P* value between groups 1 and 2, *P*2: *P* value between groups 1 and 3, *P*3: *P* value between groups 2 and 3.

Postoperative nausea and vomiting, bradycardia, and hypotension showed no significant difference between the groups, whereas itching, pruritis, and urine retention were significantly more remarkable in group 1 than in other groups (Table 8). The incidence of adverse events in group 1 was distributed as follows: nausea and vomiting (4/26), which were transient and self-limited (only 2 patients required antiemetic); hypotension (3/26), bradycardia (6/26), sedation, and dizziness (6/26); constipation (4/26, only 2 patients who needed laxative therapy); retention of urine (5/26, with only one patient catheterized); sweating (4/26); dry mouth (2/26); and itching (4/26).

**Table 8:** Side effects among the studied groups.

	Group 1 (n = 26)	Group 2 (n = 27)	Group 3 (n = 25)	*P*
PONV	4 (15.38%)	6 (22.22%)	4 (16%)	0.772
Bradycardia	6 (23.1%)	7 (25.93%)	9 (36.0%)	0.560
Hypotension	3 (11.5%)	5 (18.5%)	4 (16%)	0.776
Itching or pruritis	4 (15.38%)	0 (0%)	0 (0%)	0.019
Urine retention	5 (19.23%)	0 (0%)	0 (0%)	0.002

Data are presented as frequency (%).

PONV, postoperative nausea and vomiting.

In both ESPB groups, the following distribution of adverse events was reported: mild transient hypotension (5/27 and 4/25 in groups 2 and 3 consecutively), which was controlled by the reassurance of the patients and liberal fluid intake; PONV (6/27 and 4/25), bradycardia (7/27 and 9/25), headache (self-limited, 1/27 and 2/25); and uncomfortable thoracic hypoesthesia and numbness (2/27 and 2/25), no one of which required a reduction in the LA concentration.

## 4. Discussion

The current work compared TEA as the gold standard thoracic analgesic approach with an innovative, effective, locoregional, thoracic analgesic procedure “ESPB,” in controlling both acute and chronic PTP and their effect in reducing the emergence of chronic PTP syndrome “PTPS.” It showed that VAS values at both rest and movement were significantly higher in group 2 compared with groups 1 and 3 at 6, 24, 36 hours and at 8, and 12 weeks. The grading system for neuropathic pain was also higher in group 2 than in other groups at 8 and 12 weeks. Respectively, PTPS incidence was higher in group 2 compared with other groups at 8 and 12 weeks.

This study's results regarding the control of acute PTP and prevention of the emergence of PTPS showed that ESPB using dexmedetomidine added to local anesthetic was comparable to TEA. Both locoregional/neuraxial techniques confer multimodal analgesia, which is recommended by the American Society of Anaesthesiologists (ASA)—Task Force for managing acute postoperative pain “combined use of regional, intravenous, and oral analgesics.’^27^ The complex nature of thoracotomy pain implies the necessity of multimodal analgesia.^29^

The grading system for neuropathic pain scoring was used to detect and evaluate the neuropathic component of PTPS. It has great value in defining PTPS “as one of the postsurgical pain syndromes” and localizing the neuroanatomical mapping of traumatic injury in the somatosensory nervous system.^39^

For a long time, TEA was considered the gold standard for thoracotomy pain.^27,42,44^ Yet, TEA problems like a technical failure are high (30%), sympathectomy-associated hemodynamic liability, opioid-induced nausea, vomiting, pruritis, urinary retention, and respiratory depression, besides risks of epidural hematoma or abscesses.^35^ Furthermore, this study protocol mandates extending postoperative pain control to 5 to 6 days for managing postoperative dorsal root ganglia (DRG) hyperexcitability and neuroplasticity.^45^ Most guidelines limit epidural analgesia to 3 to 5 days to reduce the potential hazards of bacterial colonization and infection.^33^ In addition, recent recommendations were to preserve TEA/PVB for midline and bilateral thoracotomies and to reserve locoregional analgesia for unilateral thoracotomies like ESPB.^24^ Moreover, many recent reports documented permanent neurological deficits after prolonged neuraxial block, mostly claiming to direct local anesthetics neurotoxicity.^5,43^

Erector spinae plane block involves drugs injection either superficial or deep to ESM for thoracolumbar analgesia in both acute postoperative pain for thoracic surgeries, besides treating chronic and neuropathic pain conditions, eg, postmastectomy and PTP syndromes “PMPS-PTPS.”^11,15,23^ It is a novel, relatively safe, and simple technique. It usually provides effective and immediate analgesia for many thoracolumbar procedures. Ultrasonographic guidance adds more practical value for this block because it involves drug injection away from neurovascular structures and the pleura.^3,12^ Moreover, the transverse process is a sonographic landmark and backstop for needle insertion, adding to the ease, efficiency, and safety of ESPB.

Several cadaveric and multislice CT studies were performed to illustrate the spread of injectate in ESPB.^11^ It was found that solutions of local anesthetics induce sensory multidermatomal (T3 up to T11) blockade across the posterior, lateral, and anterior chest walls because of the blocking of ventral, dorsal, and lateral rami of spinal nerve roots as injectate diffuses through medial, lateral, and craniocaudal directions in the interfascial planes, through the costotransverse foramen, the intervertebral foramina, and into the paravertebral and epidural spaces. In addition, ESPB may block the rami communicans of the sympathetic chain,^11,22^ which may explain the efficacy of ESPB in relieving somatic, visceral neuropathic, and myofascial components of PTPS.^32^

The use of local anesthetic as Na-channel blockers added to the alpha 2 agonist “dexmedetomidine” in this study might eliminate the sustained peripheral input that induces abnormal central processing “central sensitization” of neuropathic component of pain.^31^ In this study, installing the drugs deep into ESP was preferred to ensure more extensive and dynamic interfacial spread craniocaudally and depositing drugs near the ventral and dorsal rami of thoracic nerves to cover multiple dermatomes. This technique is similar to other techniques described for ESPB, for example, Forero et al.^11^ Furthermore, the targeted address here is the intercostal nerve, which is the cornerstone in initiating and maintaining neuropathic PTPS due to injury of the intercostal nerve–periosteal muscle complex during thoracotomies. In addition, ESPB is better practiced in parasagittal plane close to midline at the tip of T5 transverse process to be adjacent to the costotransverse foramina, allowing efficient craniocaudal spread of injectate. More laterally, the external and internal intercostal muscles may hinder the spread.

In this trial, the authors assumed that the use of radio-opaque, wire-loaded RACZ catheter to control the direction and distance of catheter threading under brief fluoroscopic guidance and adding contrast medium to the injected cocktail to verify dermatomal levelling could add to solidity and efficacy “in the author's opinion” of ESPB in both acute postsurgical pain control (comparable VAS values to TEA) and reducing the emergence of PTPS (close to TEA values). The intermittent boluses injection is preferred over continuous infusion in the current trial and many other trials,^11,13^ which could be attributed to the opening up of the potential interfacial spaces of ESP and adding more volumes to block different rami of thoracic nerve roots that usually outlasts the duration of action of local aesthetics blockade.^3,32^

The current results showed comparable efficacy of ESPB with dexmedetomidine and TEA regarding VAS scores and less emergence of PTPS, shorter hospital stay, and fewer complications. These results are similar to the work of Forero et al.,^12^ who performed ESPB after epidural failure. Forero et al.^11^ also reported the benefits of ESPB in case series studying the treatment of PTPS and thoracic neuropathic pain.^13^ Chin et al.^3^ performed bilateral ESPB in 3 patients undergoing bariatric surgery at T7 transverse process. They postulated that efficient somatic and visceral abdominal analgesia was achieved.^3^ Tulgar et al.^40^ had documented similar visceral and somatic abdominal analgesia in laparoscopic abdominal surgery.^40^

In this regard,^36^ it was concluded that the ESPB provides similar postoperative analgesia to the TEA regarding total postoperative fentanyl requirement and pain score in pediatric patients undergoing thoracotomy. The ESPB is simpler, faster, and has a lower complication rate.

Moreover,^8^ it was reported that ESPB can be used as an effective and safe alternative to TEA for patients with lung cancer undergoing posterolateral thoracotomy.

Tulgar et al.^41^ also reported ESPB analgesia in geriatric patients with uncontrolled hypertension who underwent total hip arthroplasty. Pain relief was excellent for 2 days postoperatively, even with movement without adverse events.^41^ Erector spinae plane block reported effective analgesia for multiple rib fractures (T6–T9) even with cough and movement, resulting in rapid hospital discharge.^20^ Nagaraja et al.^27^ found that bilateral ESPB conferred lower VAS scores for 48 hours post cardiac surgery both at rest and with cough than continuous TEA with comparable ICU stay and postoperative complications. Eldemerdash and Abdelazeem et al.^7^ showed that pain relief was better with lower rescue morphine doses in ESPB than in PVB and serratus anterior plane block (SAPB) in patients who underwent breast surgeries under general anesthesia. Similar reports were documented by Bonvicini et al.^2^ who postulated that ESPB could be a safe alternative to TEA and PVB in reconstructive procedures for bilateral breast cancer. In addition, many trials suggested ESPB practice in the field of breast cancer surgeries because of its favorable values in postoperative analgesia, patient satisfaction, and opioid-sparing effect.^17,25,28,37,49^

Further multicentric meta-analysis with a higher power sample size should verify the current data. The authors suggest that adding long-acting steroids to the injected mixture “even once before catheter removal” may reduce the continuous inflammatory process that propagates into persistent postsurgical pain and spontaneous ectopic neural discharge.^31^ In addition, steroids have documented analgesic and antihyperalgesic benefits by expressing anti-inflammatory and inhibiting proinflammatory cytokines.^4^

## 5. Conclusion

Ultrasound-guided ESPB with dexmedetomidine is found to have the same potency as TEA in relieving acute post thoracotomy pain and reducing the possible emergence of chronic PTPS with a superiority to ESPB without dexmedetomidine. Erector spinae plane block has fewer side effects compared with TEA.

## Disclosures

The authors have no conflict of interest to declare.
